# Whole genome MBD-seq and RRBS analyses reveal that hypermethylation of gastrointestinal hormone receptors is associated with gastric carcinogenesis

**DOI:** 10.1038/s12276-018-0179-x

**Published:** 2018-12-03

**Authors:** Hee-Jin Kim, Tae-Wook Kang, Keeok Haam, Mirang Kim, Seon-Kyu Kim, Seon-Young Kim, Sang-Il Lee, Kyu-Sang Song, Hyun-Yong Jeong, Yong Sung Kim

**Affiliations:** 1 Genome Editing Research Center, Korea Research Institute of Bioscience and Biotechnology (KRIBB), 125 Gwahak-ro, Yuseong-gu, Daejeon 34141 Republic of Korea; 2 Personalized Genomic Medicine Research Center, Korea Research Institute of Bioscience and Biotechnology (KRIBB), 125 Gwahak-ro, Yuseong-gu, Daejeon 34141 Republic of Korea; 3 Department of Bioscience, Korea University of Science and Technology (UST), 217 Gajeong-ro, Yuseong-gu, Daejeon 34113 Republic of Korea; 4 Department of General Surgery, College of Medicine, Chungnam National University, 266 Moonwha-ro, Joong-gu, Daejeon 350157 Republic of Korea; 5 Department of Pathology, College of Medicine, Chungnam National University, 266 Moonwha-ro, Joong-gu, Daejeon 350157 Republic of Korea; 6 Internal Medicine, College of Medicine, Chungnam National University, 266 Moonwha-ro, Joong-gu, Daejeon 350157 Republic of Korea

## Abstract

DNA methylation is a regulatory mechanism in epigenetics that is frequently altered during human carcinogenesis. To detect critical methylation events associated with gastric cancer (GC), we compared three DNA methylomes from gastric mucosa (GM), intestinal metaplasia (IM), and gastric tumor (GT) cells that were microscopically dissected from an intestinal-type early gastric cancer (EGC) using methylated DNA binding domain sequencing (MBD-seq) and reduced representation bisulfite sequencing (RRBS) analysis. In this study, we focused on differentially methylated promoters (DMPs) that could be directly associated with gene expression. We detected 2,761 and 677 DMPs between the GT and GM by MBD-seq and RRBS, respectively, and for a total of 3,035 DMPs. Then, 514 (17%) of all DMPs were detected in the IM genome, which is a precancer of GC, supporting that some DMPs might represent an early event in gastric carcinogenesis. A pathway analysis of all DMPs demonstrated that 59 G protein-coupled receptor (GPCR) genes linked to the hypermethylated DMPs were significantly enriched in a neuroactive ligand–receptor interaction pathway. Furthermore, among the 59 GPCRs, six GI hormone receptor genes (*NPY1R*, *PPYR1*, *PTGDR*, *PTGER2*, *PTGER3*, and *SSTR2*) that play an inhibitory role in the secretion of gastrin or gastric acid were selected and validated as potential biomarkers for the diagnosis or prognosis of GC patients in two cohorts. These data suggest that the loss of function of gastrointestinal (GI) hormone receptors by promoter methylation may lead to gastric carcinogenesis because gastrin and gastric acid have been known to play a role in cell differentiation and carcinogenesis in the GI tract.

## Stomach cancer: DNA methylation of hormone receptor genes

A sequencing study reveals abnormal changes to DNA that set the stage for stomach cancer development. DNA methylation, the addition of methyl groups to alter DNA activity, is often disrupted in human cancers. Yong Sung Kim at the Korea Research Institute of Bioscience and Biotechnology (KRIBB) in Daejeon, South Korea, and co-workers used sequencing technogy to identify critical methylation changes in stomach epithelial cells, intestinal metaplasia lesion and tumor cells during early-stage gastric cancer. The team found 3,035 abnormally methylated DNA regions related to the expression of particular genes. Further analysis identified six hormone receptor genes directly involved with stomach acid secretion, whose altered expression was linked to over-methylated DNA regions. Loss of function within these six genes may lead to gastric cancer, and their expression levels could be valuable biomarkers for the disease.

## Introduction

Gastric cancer (GC) remains the second most frequent cause of death from cancer in both sexes worldwide^1^, although considerable progress has been achieved in developing early detection approaches and improving surgical procedures and adjuvant chemotherapy^2,3^. Most importantly, the precise mechanisms underlying gastric carcinogenesis and disease progression are not fully understood. GCs can be divided into two distinct histological groups, i.e., the intestinal and diffuse types^4^. Intestinal-type GCs (IGCs) are histologically differentiated and develop through the following well-characterized sequential stages as precancerous lesions: chronic gastritis, atrophy, intestinal metaplasia (IM), and dysplasia. In contrast, diffuse-type GCs (DGCs) are histologically undifferentiated and develop through a shorter, less characterized sequence of events from gastric epithelial cells^5^.

Previous studies have shown that *H. pylori* infection causes aberrant DNA methylation in gastric epithelial cells and induces remarkable inflammation^6^, indicating that epigenetic alterations are potentially some of the earliest abnormalities during gastric carcinogenesis. Currently, epigenetic changes, such as the hypermethylation of tumor suppressor genes and the hypomethylation of oncogenes, are considered hallmarks of cancer that play a key role in the development and maintenance of the malignant phenotype^7–9^.

To detect differences in critical epigenetic signatures between paired normal and tumor tissues, it is necessary to extract a microscopic homogeneous cellular subpopulation from its complex tissue milieu^10^. However, the reliability of tests based on tissue often crucially depends on the relative abundance of the cell population in question^5^. Therefore, a prerequisite for modern molecular research is the preparation of pure samples without a large number of “contaminating” cells^11,12^. Laser capture microdissection (LCM) offers a simple single-step process that can be used as a rapid and dependable method of preserving and isolating clusters of cells from tissue sections by direct microscopic visualization^10,13,14^.

The aim of this study was to reveal sequential changes in DNA methylation in gastric carcinogenesis and identify the critical pathway associated with GC development. In this study, we combined methylated DNA binding domain sequencing (MBD-seq) with reduced representation bisulfite sequencing (RRBS) to perform a comprehensive methylome analysis in gastric mucosa (GM), IM, and gastric tumor (GT) cells isolated from a patient with IGC by LCM. Here, we provide information regarding the hyper- and hypomethylation signatures in IM and GT cells during intestinal-type gastric carcinogenesis and show that gastrointestinal (GI) hormone receptor genes in a neuroactive ligand-receptor interaction pathway are predominantly hypermethylated in GCs. To the best of our knowledge, this study is the first to identify a unique pathway associated with human disease based on a methylome analysis.

## Materials and methods

### Cell lines and tissue samples

The GC cell lines (SNU-001, SNU-005, SNU-016, SNU-216, SNU-484, SNU-520, SNU-601, SNU-620, SNU-638, SNU-668, SNU-719, AGS, KATOIII, MKN1, MKN45, and MKN74) were obtained from the Korean Cell Line Bank (http://cellbank.snu.ac.kr). The paired primary GC tissues and adjacent normal gastric tissues from 175 patients with gastric cancers were obtained with informed consent from the BioBank of Chungnam National University Hospital, Daejeon, Korea, and their use was approved by the Institutional Review Board of the Hospital. The cancer tissues were histologically confirmed by a pathologist, and the clinical information was obtained from the medical records.

### Laser capture microdissection

Fresh untreated specimen from the stomach of a patient with early gastric cancer (EGC) was obtained by endoscopic submucosal dissection (ESD) and embedded in Tissue-Tek OCT medium (Sakura, Tokyo, Japan). Using a cryostat (Microtome, Leica, Germany), ten serial sections (8 μm thick) of the frozen specimen were cut onto PALM Membrane Slide 1.0 PEN slides (Zeiss Microimaging, Munich, Germany), stained with H&E and then coated using Liquid Cover Glass N (Carl Zeiss, Germany) for image enhancement and sample protection. The GM, IM, and GT cells were delineated using the PALM Robosoftware (Zeiss Microimaging) and cut into 0.5-mL Adhesive-Cap tubes using a PALM Laser capture microdissection system (Zeiss Microimaging). The genomic DNAs of the captured cells were obtained using a QIAamp DNA Micro Kit (Qiagen, Valencia, CA). The genomic DNA was subjected to electrophoresis on a 0.8% agarose gel; then, the gel was stained with GelRed (Biotium, Fremont, CA), and the DNA concentration was quantified using a PicoGreen dsDNA Quantitation Kit (Molecular Probe, Eugene, OR).

### Estimation of tumor cell content in GT cells isolated by LCM

To examine the cell homogeneity in the biopsy sample isolated by LCM, we estimated the content of the tumor cells in the GT cells isolated by LCM through multiple displacement amplification (MDA) of LCM-DNA, a copy number of variation (CNV) analysis, and a pyrosequencing analysis for genotyping at selected loss of heterozygosity (LOH) loci. These procedures are described in Supplementary online material.

### MBD-seq analysis

For the MBD-seq analysis, LCM-DNAs from GM, IM, and GT cells were fragmented to 100–500 bp by 44 psi of gas for 1 min through a nebulizer (Illumina, San Diego, CA) and then subjected to methylated DNA enrichment using a MethylMiner Methylated DNA Enrichment Kit (Invitrogen, Carlsbad, CA). Briefly, the methylated DNAs were precipitated from each 500 ng of fragmented LCM-DNAs via binding to the methyl-CpG binding domain of`the human MBD2 protein, which was coupled to magnetic Dynabeads. Then, the methylated fragments were eluted with High-Salt Elution Buffer (Invitrogen) and purified with a MinElute PCR Purification Kit (Qiagen). The methylated DNA fragments were ligated to a pair of adaptors for Illumina sequencing. The ligation products were size-fractioned on a 2% agarose gel to obtain 200–300 bp fragments and subjected to 18 cycles of PCR amplification. Each library was diluted to 8 pM, and 76 cycles of single-read sequencing was performed on an Illumina Genome Analyzer II.

### RRBS analysis

RRBS was performed as previously described^15^ using each 300 ng of LCM-DNA (GM, IM, and GT) as input. The experimental protocol steps were as follows: (i) DNA digestion using the *Msp*I restriction enzyme, which cuts DNA at its recognition site (C↓CGG) independent of the CpG methylation status; (ii) end repair and ligation of adapters for Illumina sequencing; (iii) gel-based selection of DNA fragments with insert sizes ranging from 40 bp to 120 bp and 120–220 bp; (iv) two successive rounds of bisulfite treatment, after which we observed 98% converted cytosines outside the CpGs; (v) 18 cycles of the PCR amplification of the bisulfite-converted library; and (vi) 76 cycles of single-read sequencing using an Illumina Genome Analyzer II.

### Base-calling and mapping

For the MBD-seq, base-calling was performed throughout the routine process of the Illumina pipeline module bclConverter v1.7 during the 76 single-read cycles. The sequences were aligned to the human genome assembly (hg18) using ELAND version 2 with the default parameters. To evaluate the methylation peak signatures, the aligned coordinated sequences were extended up to 200 bp from the start position. The mapped reads (76 bp) are partial fragments of sonicated DNA after size selection (200 bp). The coverage depth of the methylated reads was counted per 200 bp resolution. The calculated count value was converted into a methylation enrichment score (MES) to remove bias among the number of reads from the different samples. The raw signals (n) in each bin size resolution were transformed into an adjusted value (MES) based on the ratio of the total number of reads (total n) to the genomic size (L) using the following formula:

$${\mathrm{MES}}_{{\mathrm{bin}}_{\mathrm{i}}} = \log \left( {\frac{{{\mathrm{n}}/200({\mathrm{bin}}_{\mathrm{i}})}}{{{\mathrm{total}}\,{\mathrm{n}}/{\mathrm{L}}}}} \right)$$

The adjusted MES signals were exported in a BED file format and visualized with our lab mirror of the UCSC Genome Browser due to the large size.

For the RRBS analysis, the base calling process was the same as that performed for the MBD-seq. However, the mapping step was performed with the BRAT software, which is a methylation specific mapping tool^16^ for short bisulfite-treated reads because the unmethylated cytosines in the sequenced reads from RRBS are converted into thymines. The methylation value of each CpG site was calculated as ‘T’ (unmethylation) and ‘C’ (methylation) read counts using the pysam Python package.

### Efficiency of sodium bisulfite conversion

The bisulfite conversion efficiency of our sequence data mapping was calculated with the BRAT^16^ tool. More than 98% of cytosines, excluding CpG sites, were converted into thymine residues based on the reference genome.

### Coverage and depth analysis

MBD-seq is sensitive to highly methylated regions that have high CpG densities with different genomic features. Thus, the coverage and depth of the uniquely mapped reads were calculated against the total number of 200 bp bins with a C, G, or CpG context as a reference ^17,18^. The coverage and depth of four genomic regions, including the whole genome, promoters, CpG islands (CGIs), and intergenic region, were estimated. A promoter region was defined as 2 kb centered on a TSS of RefSeq and CGI information and was obtained from the UCSC Genome Browser website. The coverage and depth of the RRBS analysis were calculated using the same procedure described for MBD-seq, except for the methylated abundance at all CpG sites that are not C or G and are not consecutive was addressed.

### Identification of differentially methylated regions

The sliding-window approach was applied to identify differentially methylated regions (DMRs) with methylation differences greater than 2-fold between the samples (GM vs. IM or GM vs. GT) within a 1 kb tile per 200 bp bin shift (*t*-test, *P* < 0.01) in the MBD-seq data. Hyper- or hypomethylated DMRs indicate DNA regions that are methylated by more or less than 2-fold in the GT or IM compared to those in the GM. We defined “early-onset DMRs” as DMRs commonly observed in the IM and GT compared to the GM and “GT-specific DMRs” as DMRs observed in the GT but not the IM compared to the GM. In the RRBS data, the methylated and unmethylated CpGs were counted, and Fisher’s exact test was used to determine the methylation frequency during gastric carcinogenesis using a 2 by 3 contingency table. After the frequency test, Bonferroni correction criteria were applied to all CpG sites sequenced by RRBS to restrict type 1 errors. Then, the regions showing methylation differences with more than 20% changes between the samples (GM vs. IM or GM vs. GT) at a CpG site were selected as DMRs for RRBS. The DMRs in the promoter regions from the MBD-seq data were defined as differentially methylated promoters (DMPs), and promoter regions with three or more significant CpG sites from the RRBS data were defined as DMPs for RRBS.

### Influence of DMRs on genomic features

To infer the biological significance of the DMRs on the genomic features, the distributions of the DMRs from the MBD-seq and RRBS data were compared in terms of the following four genomic features: (i) whole genome, (ii) promoters (CGI-associated, shore, shelf, and non-CGI-associated), (iii) intragenic (exons, except for the first exon, introns, 3′UTRs, repeat elements and CGIs), and (iv) intergenic regions (repeat elements and CGIs) based on the UCSC website (hg18). In the promoter regions, regions 0–2 kb or 2–4 kb upstream of the CGIs were defined as CGI shores or CGI shelves, respectively, as previously described^16^.

### Pathway enrichment analysis

A KEGG pathway enrichment analysis was conducted using the Functional Annotation tool in DAVID Bioinformatics Resources^19^. Gene sets linked to hypermethylated or hypomethylated DMPs were used as input in DAVID for the mining of the functional relevance of the methylation changes.

### RT-PCR and real-time quantitative RT-PCR

RT-PCR and qRT-PCR analyses were performed to validate the expression levels of genes identified as methylated targets in the GC cell lines and clinical tissues as previously described^20^. The primer sequences are listed in Supplementary Table S12. The PCR conditions were as follows: 94 °C for 5 min, 25–35 cycles at 94 °C for 30 s, annealing temperature (60–68 °C) for 30 s, 72 °C for 30 s and a final cycle at 72 °C for 7 min. The RT-PCR products were analyzed on 1.5% agarose gels stained with ethidium bromide. The *β-actin* gene was used as a control. The cDNA (100 ng) was amplified by 45 cycles with 2 × SYBR Green Supermix (Bio- Rad, Hercules, CA) using the primer sets. Real-time qRT-PCR was performed using a C1000 Thermal Cycler (Bio-Rad, Hercules, CA, USA). The gene encoding *β-actin* was amplified as a control. The relative quantification of the target mRNAs was performed using the comparative threshold cycle (Ct) methods.

### Treatment of GC cells with 5-aza-2′-deoxycytidine and trichostatin A

GC cell lines, including SNU-216, SNU-484, SNU-638, and MKN1, were seeded at a density of 1 × 10^6^ cells per 10-cm dish and cultured for 1 d before the drug treatment. The cells were treated with 10 μmol/L 5-aza-2′-deoxycytidine (AZA; Sigma) every 24 h for 3 days and then harvested. Another culture of cells was treated with 250 nmol/L trichostatin A (TSA; Sigma) for 1 day and then harvested. To test the synergistic effects of AZA and TSA, the cells were first treated with 10 μmol/L AZA for 3 days, followed by treatment with 500 nmol/L TSA for 1 day. The total RNA was prepared, and the effect on the target expression was assessed by real-time qRT-PCR.

### Bisulfite sequencing analysis

Genomic DNA (2 μg) from the GC cell lines or clinical tissues was modified with sodium bisulfite for 16 h using an EZ DNA Methylation Kit (ZYMO Research, Orange, CA). The bisulfite-modified DNA was amplified using primer sets designed to amplify the regions of interest. The PCR primer sequences used for the bisulfite sequencing were designed by MethPrimer (http://www.urogene.org/methprimer/index1.html; see Supplementary Table S12). The amplification was performed using the following conditions: initial denaturation step at 95 °C for 10 min; 35 cycles of denaturation at 95 °C for 45 s, 60–63 °C for 45 s, and 72 °C for 1 min; and a final cycle at 72 °C for 10 min. The PCR products were purified from a gel using a Qiagen Gel Extraction Kit (Qiagen, Valencia, CA) and cloned into a pGEM-T Easy Vector (Promega, Madison, WI) for sequencing. Five to ten clones were selected for sequencing. The methylation percentage of each sample was calculated as the number of methylated CpG dinucleotides from the total number of CpGs.

### Risk score development

To develop an easy-to-use risk score for patients, a previously developed strategy using a Cox regression coefficient for several genes from patient cohorts was adopted^21,22^. The risk score of each patient was calculated as the sum of each gene’s score, which was calculated by multiplying the expression level of the gene by its corresponding coefficient using the following formula: Risk score = ∑ Cox coefficient of gene *G*_*i*_ × expression value of gene *G*_*i*_. Then, the patients were divided into two groups (i.e., high- or low-risk of survival) using the median cut-off of the risk score as a threshold. The differences in survival between the patient groups were estimated by the Kaplan–Meier method and a log-rank test.

### Public data

Expression and 450 K HumanMethylation array data for GCs were downloaded from The Cancer Genome Atlas (TCGA) data portal (https://portal.gdc.cancer.gov/). We obtained expression and methylome data of primary GCs and normal tissues. The CGI coordinates were obtained from the UCSC browser. The CGI shores and shelves were obtained from the CGI coordinates by considering the 0–2 kb and 2–4 kb regions upstream of the CGI flanking regions.

## Results

### Isolation of highly homogeneous cell clusters using LCM

**Fig. 1:**
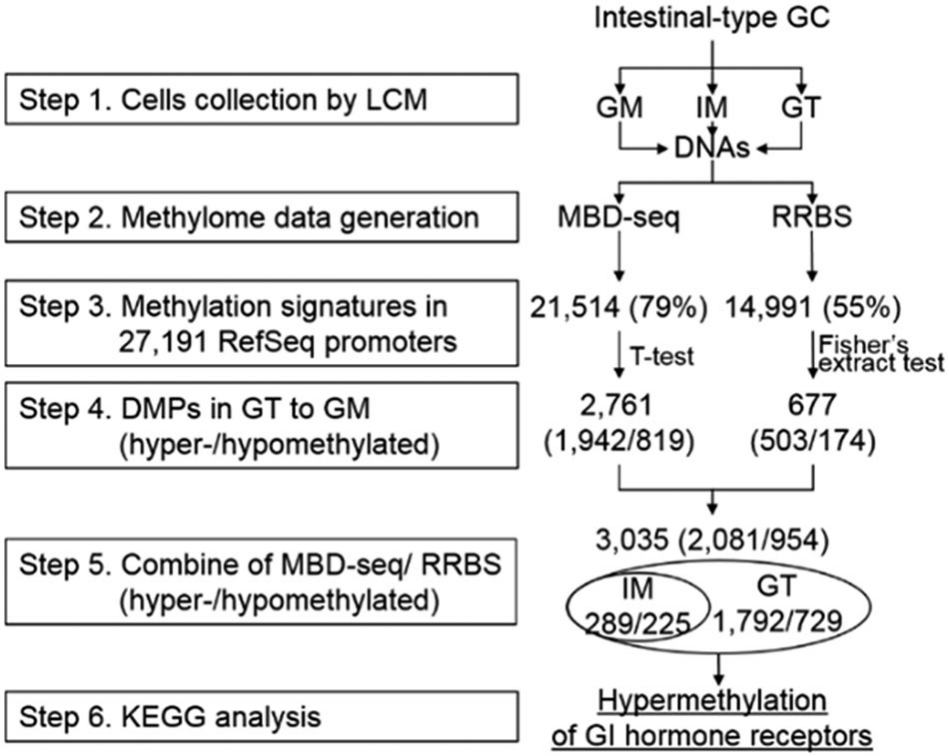
Schematic diagram of DNA methylation profiling of gastric carcinogenesis using MBD-seq and RRBS. The six-step process of the initial methylome profiling, identification of the promoter DMRs, and pathway analysis associated with gastric carcinogenesis

The overall six-step process of this study is summarized in Fig. 1. We performed a methylome analysis of ESD tissue from a patient with EGC. To procure highly homogenous tissue cells, we isolated approximately 4,000 crypt-containing GT cells (1.1 × 10^5^ cells) and 4000 crypt-containing GM and IM cells (1.2–1.3 × 10^5^ cells) from frozen ESD tissue using an LCM procedure (Step 1 in Fig. 1 and Supplementary Fig. S1). We obtained 0.86–1.16 μg of DNA from each isolated cell (Supplementary Table S1), and gel electrophoresis was performed to show the high molecular weight DNAs in all cell types (Supplementary Fig. S2a).

To test the cell homogeneity, we performed a CNV analysis of the GM, IM, and GT DNAs using an Affymetrix SNP 6.0 array. The GM and IM DNAs did not show changes in the copy number compared to a reference set, while the GT cells harbored copy number gains in eight chromosomal regions and losses in six chromosomal regions (Supplementary Fig. S3a–c and Supplementary Table S2).

Based on the copy number loss data, we selected five LOH loci that were presented as heterozygous alleles in the GM cells, although one allele was missing in the GT cells. The cell homogeneity test showed that the average frequencies of the A allele in the five loci were 0.56 and 0.66 in the DNA from the bulk normal and tumor tissues and 0.53 and 0.94 in the DNA from the GM and GT cells isolated by LCM, respectively (Supplementary Fig. S4). These results indicate that the LCM procedure isolated cell clusters with high homogeneity from the tissue sections based on the tumor cell contents.

### Methylome profiling of GM, IM, and GT cells using MBD-seq and RRBS

**Fig. 2:**
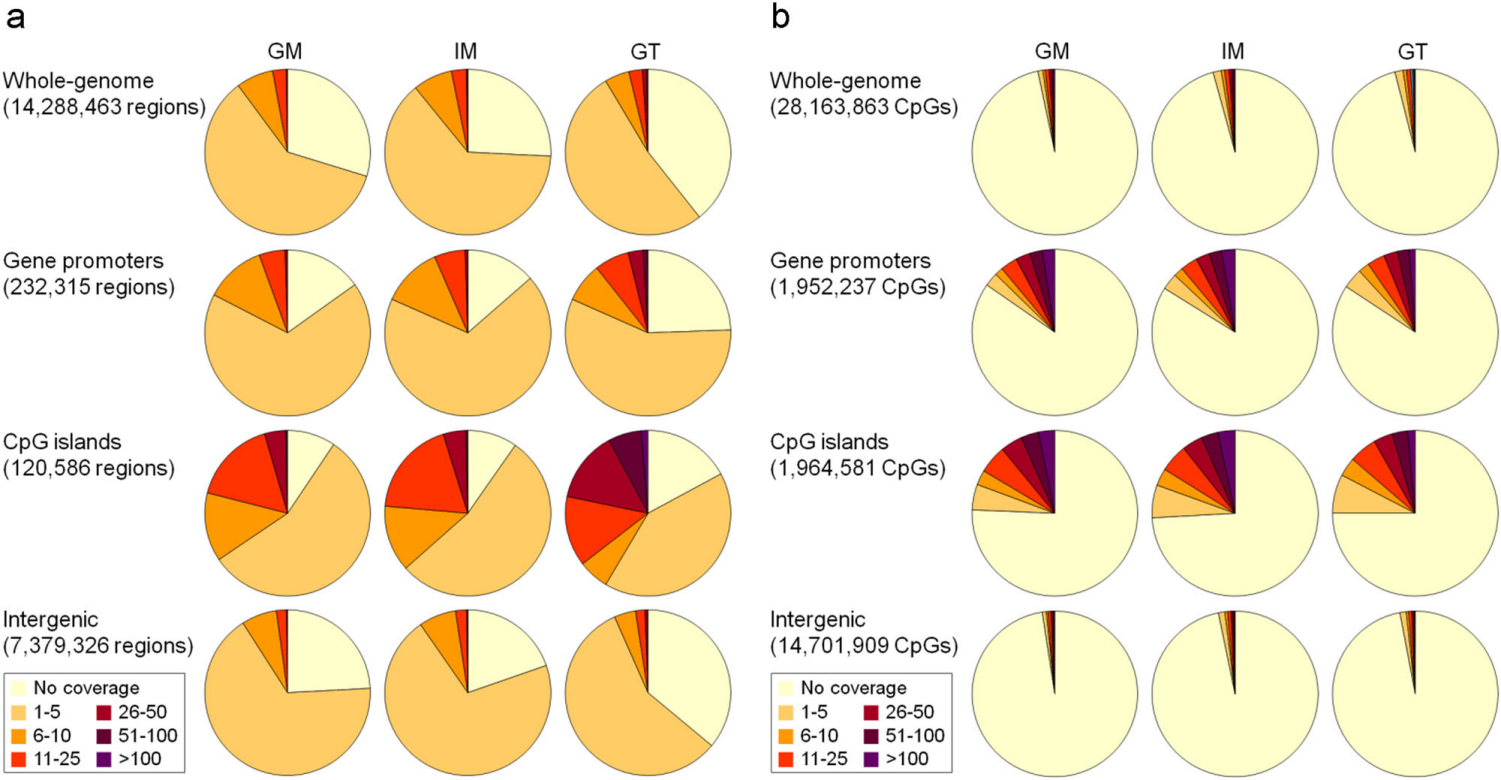
Genome coverage of the methylation signatures from the MBD-seq and RRBS data of samples from a patient with GC. (**a**) For the MBD-seq analysis, the methylation enrichment signatures were searched by 200 bp sliding on the whole-genome, gene promoters (2 kb regions centered on TSSs from RefSeq), CGIs, and intergenic (intergenic regions without promoters) regions. (**b**) For the RRBS analysis, the genomic coverage was assessed at the single base level in the same regions as those included in the MBD-seq analysis

As shown in Fig. 1, in Step 2, we established libraries for the MBD-seq and RRBS analyses using 500 ng and 300 ng of LCM-isolated DNA, respectively (Supplementary Fig. S2b and S2c). A Genetic Analyzer II generated 24.3, 28.3, and 24.6 million reads for the GM, IM, and GT MBD-seq libraries, and 70.4, 64.7, and 69.9% of the reads were aligned to hg18, respectively (Supplementary Table S3). The MBD-seq reads covered approximately 68% of the 14,288,463 segments (200 bp bins) in the human whole-genome, 82% of the 232,315 segments in the promoters, 88% of the 120,586 segments in the CGIs, and 73% of the 7,379,326 segments in the intergenic regions (Fig. 2a) Highly covered regions with a read depth ranging from 26–100 were significantly increased in the promoter and CGI regions of the GT cells compared to those in the GM and IM cells. The coverage distribution was very similar between the GM and IM cells.

In contrast, the number of sequencing reads from the RRBS libraries was estimated to be half of the number of reads from MBD-seq, including 13.0, 18.9, and 13.4 million reads in the GM, IM, and GT libraries, and 62.4, 37.9, and 40% of the reads were aligned to hg18, respectively (Supplementary Table S3). The DNA sequences covered approximately 3.9% of the 28,163,863 CpG sites in the human whole-genome, 15.7% of the 1,952,237 CpG sites in the promoters, 25.1% of the 1,964,581 CpG sites in the CGIs, and 2.9% of the 14,701,909 CpG sites in the intergenic regions (Fig. 2b). These data indicate that the RRBS procedure enriched genomic regions (selectivity by MspI), such as CGIs or gene promoters, and that the distribution patterns appear to be relatively similar among the three cell types (Fig. 2b). The methylation signatures in the MBD-seq and RRBS data matched 21,514 (79%) and 14,991 (55%) of the 27,191 RefSeq promoters predicted in hg18, respectively.

### Identification of DMRs in the MBD-seq and RRBS data

**Fig. 3:**
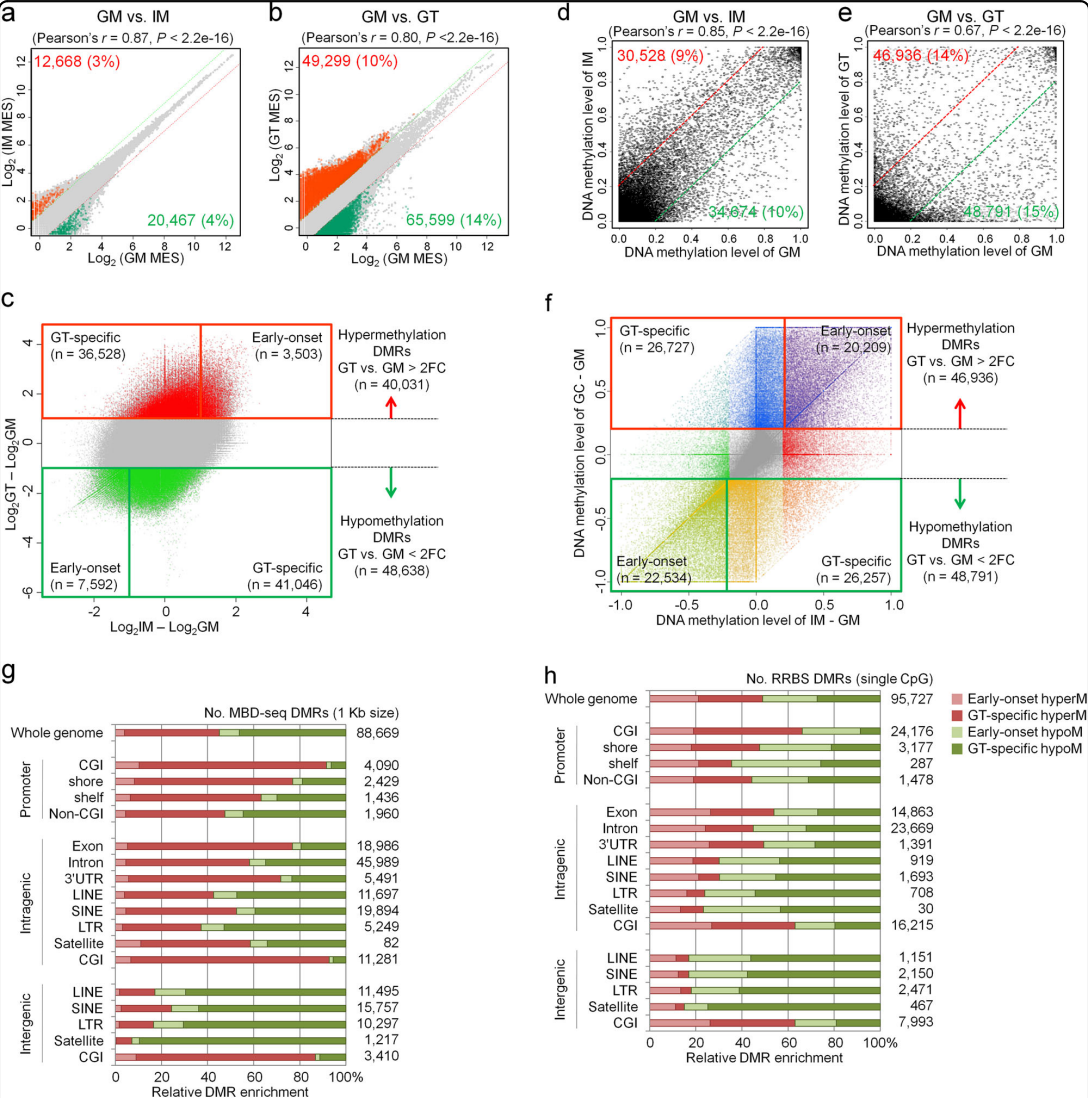
Global comparison of three methylome datasets from MBD-seq and RRBS analyses of samples from a patient with GC. **a**, **b** For the MBD-seq data, a pairwise correlation of DNA methylation in 1 kb tiles was performed. The genomic tiling was obtained by sliding a 1 kb window through the genome such that each tile starts at the position where the previous tile moves 200 bp down. The average methylation value of each 1 kb tile was calculated, and 479,941 1-kb tiles had an average methylation value greater than 10 in at least one of the three cell types. Red or green spots indicate significant 2-fold increases (hypermethylation) or decreases (hypomethylation) between the data sets. **c** Two-dimensional scatter plot of the MBD-seq data. **d**, **e** For the RRBS data, a pairwise correlation of the DNA methylation data at individual CpG sites was performed. Red or green lines indicate 20% increased methylation levels (hypermethylation) or 20% decreased methylation levels (hypomethylation) between the cell types. **f** Two-dimensional scatter plot of the RRBS data. **g**, **h** Genomic distribution of DMRs according to the genomic features in the MBD-seq and RRBS data

We found unusual methylation enrichment patterns, such as mono-, tri-, or tetra-ploidy, in the aneuploidy chromosomes of the GT cells, highlighting the direct correlation between methylation enrichment and chromosomal aneuploidy. To account for the effects of aneuploidy on methylation enrichment, we normalized the read counts from the GT cell MBD-seq data considering the CNV effect (Supplementary Fig. S5). Subsequently, we performed scatter plot analyses to determine the methylation pattern differences between the IM and GT cells and the GM cells. For the MBD-seq data, the average methylation values were counted within a 1 kb tile and spotted for IM vs. GM (Fig. 3a) or GT vs. GM (Fig. 3b). The hyper- ( > 2-fold) or hypomethylated ( < 2-fold) signatures in the IM vs. GM cells (*t*-test, *P* < 0.01) accounted for 3 or 4% of all spots (479,941), respectively, (Fig. 3a). The hyper- or hypomethylated signatures in the GT vs. GM cells accounted for 10 or 14% of all spots (479,941), respectively (Fig. 3b).

To identify the DMRs between the IM and GT cells and the GM cells, the methylation values between the IM and GM and between the GT and GM were spotted two-dimensionally (Fig. 3c). First, we identified 40,031 DMRs with methylation differences > 2-fold change between the GT cells and the GM cells on the *y*-axis (Fig. 3c, Supplementary Table S4). These DMRs were divided into two subsets as follows: 36,528 ‘GT-specific’ and 3503 ‘early-onset’ hypermethylated DMRs with methylation differences  > 2-fold change between the IM cells and GM cells on the *x*-axis. Similarly, 48,638 hypomethylated DMRs exhibited methylation differences  < 2-fold change between the GT cells and GM cells on the *y*-axis, including 41,046 DMRs classified as GT-specific and 7,592 DMRs classified as early-onset hypomethylated DMRs (Supplementary Table S4 and Fig. 3c).

In the RRBS analysis, we compared the DNA methylomes of the two different cell types at 332,448 CpG sites with a minimum read depth of 10 using scatter plots and observed significant *Pearson*’s correlations between the IM and GM cells (*R* *=* 0.85, *P* < 2.2 × 10^−16^) (Fig. 3d) and between the GT and GM cells (*R* *=* 0.67, *P* < 2.2 × 10^−16^) (Fig. 3e). In contrast to the MBD-seq analysis, the methylation in the RRBS analysis was unaffected by the CNV data. Thus, we directly identified 95,727 DMRs with methylation differences > 20% changes between the samples at a single CpG site. These DMRs included 46,936 hypermethylated DMRs (20,209 early-onset and 26,727 GT-specific) and 48,791 hypomethylated DMRs (22,534 early-onset and 26,257 GT-specific) (Fig. 3f).

### Global distribution of DMRs in the human genome

Based on our both methylome data, we performed a relative enrichment analysis based on the number of DMRs in each genomic feature group (see Materials and methods section) to identify where methylation events frequently occur in the GT and IM cell genomes. On the whole genome level, the number and distribution of hypo- and hypermethylated DMRs (48,638 and 40,031) in the MBD-seq data (Fig. 3g) in the GT cells did not different from those (48,791 and 46,936) in the RRBS data (Fig. 3h), but the proportion of early-onset DMRs in the RRBS data was larger than that in the MBD-seq data. However, in the CGI-associated promoters in the GT cells, 92% (3,745 of 4,090) of the DMRs were hypermethylated in the MBD-seq data, while only 66% (15,989 of 24,176) of the DMRs were hypermethylated in the RRBS data, suggesting that CGI hypermethylation at promoter regions may be inconsistent using the two methods partially due to the methods’ properties.

Both sets of methylome data showed a similar distribution of hypo- and hypermethylated DMRs in the intergenic region. For example, in the GT cell genome, we found that repeat elements, such as LINEs (83%), SINEs (76%), LTRs (83%), and satellites (93%), are predominantly hypomethylated in intergenic regions in the MBD-seq data, although they display balanced hypermethylation and hypomethylation in intragenic regions (Fig. 3g). This finding indicates that the intergenic repeat elements are heavily methylated in the GM cell genome but become demethylated in GT cells. Thus, these data indicate that the epigenetic mechanism repressing the activity of transposable elements, such as LINEs, can be disrupted by demethylation in cancer cells, which facilitates mutagenic retrotranspositions. The most statistically significant changes in repeat elements were observed in satellite DNAs in intergenic regions, suggesting that satellite DNA hypomethylation may participate in the development of GC by inducing aneuploidy.

### Identification of DMPs based on the RRBS and MBD-seq analyses

To identify epigenetic targets associated with gastric carcinogenesis, we focused on the DMPs, which were defined as DMRs in promoter regions based on both the MBD-seq and RRBS data. Based on the hyper- and hypomethylated DMRs (Supplementary Table S4) detected in the MBD-seq data, 1,942 hyper- (Supplementary Table S5) and 819 hypomethylated DMPs were identified (Supplementary Table S6).

The RRBS procedure is enriched in genomic regions with high densities of CpGs, such as gene promoters (Fig. 2b); thus, we directly identified DMRs in the promoter regions covered by the RRBS reads between GT cells and GM cells and defined these DMPs in the RRBS analysis as three or more DMRs located in a promoter region. Of the 14,991 promoters covered by the RRBS reads (Step 3 in Fig. 1), we selected 677 DMPs that significantly differed between the GM and IM cells and/or GT cells (Supplementary Table S7) based on observations by two investigators of each track on the genome browser with the naked eye. The DMPs could be divided into 503 hyper- (Supplementary Table S8) and 174 hypomethylated DMPs (Supplementary Table S9) (Step 4 in Fig. 1).

The different attributes of the MBD-seq and RRBS technologies may lead to discrepancies regarding the presence of methylation signatures in CpG sequences. Nevertheless, we found that many (403) DMPs were detected in both analyses, indicating consistency between the two technologies. However, we expect that the two different approaches may be mutually complementary and thereby provide information regarding useful targets that a single technology may not be able to detect. Thus, in Step 5 shown in Fig. 1, we combined the DMPs from the MBD-seq and RRBS analyses and identified a total of 3,035 DMPs, including 2081 hypermethylated and 954 hypomethylated DMPs. The DMPs were classified as 514 early-onset and 2,521 GT-specific DMPs (Table S10 and Supplementary Table S11).

### Pathway enrichment analysis of DMPs

**Fig. 4:**
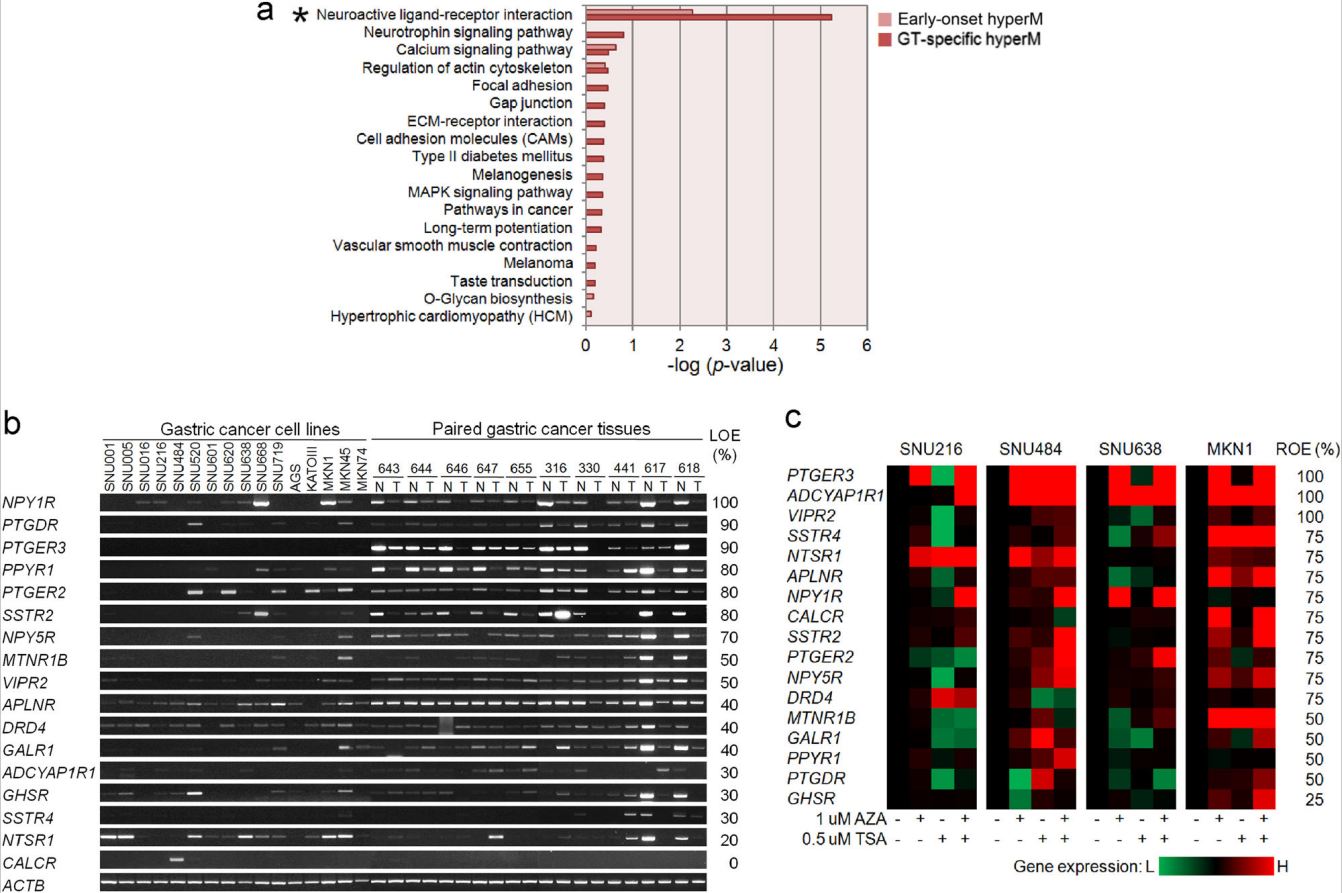
Pathway enrichment analysis of putative genes correlated with DMPs and expression analysis of genes associated with the neuroactive ligand-receptor interaction pathway. **a** Pathway enrichment analysis of hypermethylated genes in GC. The *x*-axis shows −log (Benjamini test, *P*), and the *y*-axis shows the pathway categories. Stars indicate significant pathway categories. **b** RT-PCR analysis of 16 GC cell lines and ten paired gastric tumor (T) and normal match control (N) tissues. The LOE column indicates the loss of expression as percentages of gene expression upregulation in gastric tumors compared to that in normal tissue. **c** Restoration test of gene expression in 17 genes in four GC cell lines following treatment with the DNA methylation inhibitor AZA and/or TSA treatment. ROE indicates restoration of expression as a percentage of a GC cell line in which expression was restored

To infer the functional role of the identified DMPs, we performed a pathway enrichment analysis of a gene set linked to the 3,035 DMPs using DAVID, which is a web-based tool developed for Gene Ontology ranking (Step 6 in Fig. 1). We identified 59 G protein-coupled receptor (GPCR) genes linked to the hypermethylated DMPs, including 13 GPCRs associated with early-onset DMRs and 46 GPCRs associated with GT-specific DMRs, that were significantly enriched in a neuroactive ligand-receptor interaction pathway (Benjamini test, *P* = 0.005 in the early-onset group; *P* = 5.78 × 10^−6^ in the GT-specific group) (Fig. 4a, Table 1).

**Table 1:** Pathway enrichment analysis of all genes selected from the MBD-seq and RRBS analyses

KEGG pathway	Matched	Characteristics	Benjamini^a^	Gene list
Neuroactive ligand-receptor interaction (256 genes)	13	Early-onset, hypermethylated	0.005	*SSTR4, EDNRB, DRD1, GRIK1, GRIK2, CHRM2, NPBWR1, PTGDR, GABRA5, ADRA1A, GHSR, HTR2C, GABRQ*
Neuroactive ligand-receptor interaction (256 genes)	46	GT-specific, hypermethylated	5.78 × 10^−6^	*CALCR, TACR3, GABRB3, GRIK3, ADCYAP1R1, LEPR, LHCGR, PPYR1, DRD4, LPAR3, VIPR2, EDNRA, APLNR, HTR1B, HTR1A, GALR1, P2RY4, LTB4R, GRID1, PTGER2, GLRB, PTGER3, PTH2R, OPRL1, GRIN1, NPY1R, GRIA4, NTSR1, GRM1, NPY5R, CRHR2, SSTR2, P2RY11, GRM2, CHRM4, ADRB1, GRIA2, GRM7, MLNR, LTB4R2, P2RX2, F2, GRM6, MTNR1B, GPR50, UTS2R*

^a^Significant pathway was examined by Benjamini test (*P* < 0.05)

### Hypermethylation of GI hormone receptors in the stomach

Of the 59 GPCRs, we selected 17 GI hormone receptor genes related to the regulation of gastric acid secretion and/or gastric injury healing and validated their gene expression levels in 16 GC cell lines and 10 paired primary GC tissues. Most genes were not found to be expressed or downregulated in most tested GC cell lines (Fig. 4b). The expression of nine genes was down-regulated in over 50% of primary gastric tumors compared with that in the paired normal tissues (Fig. 4b).

The treatment with AZA and/or TSA in four GC cell lines restored the expression of the 17 GI hormone receptor genes in at least one of the GC cell lines tested, indicating that the expression of these genes might be controlled by epigenetic mechanisms (Fig.4c). Among these genes, we further selected six GI hormone receptors whose expression was significantly down-regulated by 80–100% in the primary gastric tumors, including neuropeptide Y receptors *(NPY1R* and *PPYR1)*, prostanoid receptors (*PTGDR*, *PTGER2*, and *PTGER3)*, and a somatostatin receptor (*SSTR2)* (Fig. 4b).

### Validation of the expression and methylation of the six GI hormone receptors

**Fig. 5:**
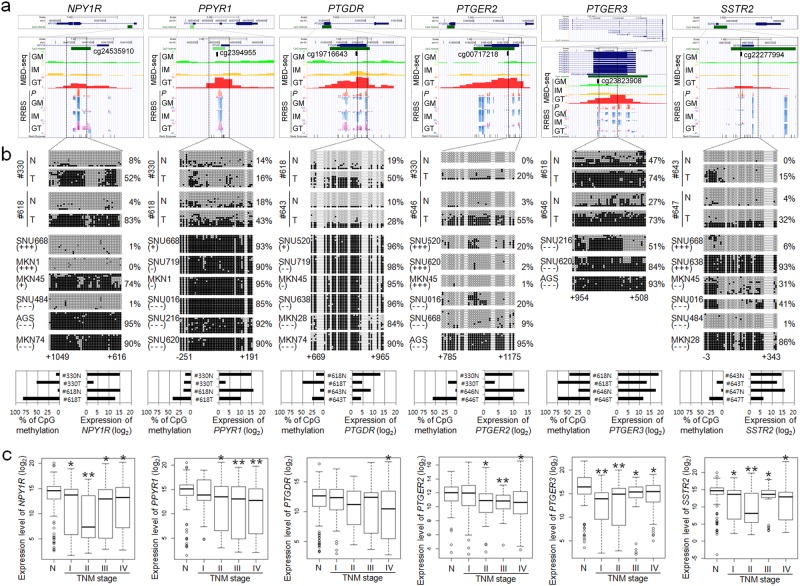
Methylation and expression analysis of six hypermethylated genes. **a** Methylation signatures at the promoter regions of the six hypermethylated genes from the MBD-seq and RRBS data. **b** Comparison of promoter methylation and expression of each gene in two sets of paired gastric tumor tissues. Promoter methylation was estimated by bisulfite sequencing, and expression was estimated with a real-time qRT-PCR analysis. **c** Comparison of the expression levels of the six genes between gastric tumor and normal tissues in the CNUH cohort using a real-time qRT-PCR analysis. All analyses were performed by Student’s paired *t*-test

Using Genome Browser, we confirmed that the CGIs in the promoter regions of the six GI hormone receptors were heavily methylated in the GT cells compared with those in the GM and/or IM cells and that the methylation status of each target corresponded well between the MBD-seq and RRBS data (Fig. 5a). The qRT-PCR and bisulfite sequencing analyses of the six genes *(NPY1R, PPYR1, PTGDR*, *PTGER2*, *PTGER3*, and *SSTR2)* demonstrated a negative correlation between methylation and gene expression in two sets of paired gastric tumor and normal tissues (Fig. 5b).

**Fig. 6:**
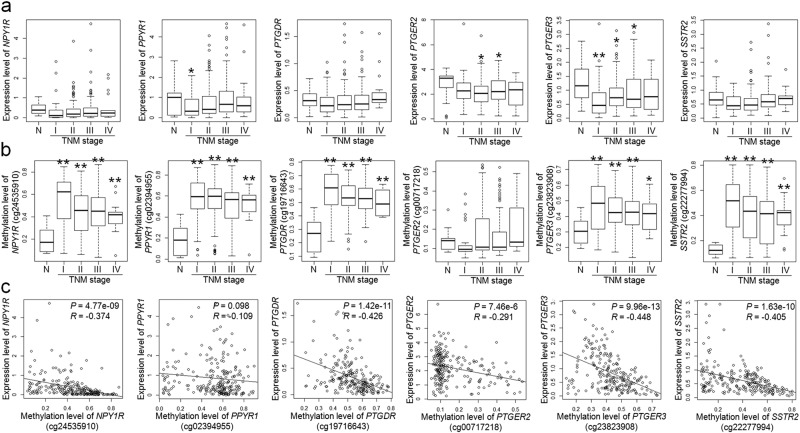
Methylation and expression analysis of six hypermethylated genes in the TCGA database. **a** Expression analysis of each gene in gastric normal tissues (*n* = 29) and gastric tumors by TNM stages (stage I, *n* = 47; II, *n* = 108; III, *n* = 99; and IV, *n* = 18) from TCGA database using RNA-seq. **b** Methylation analysis of each gene in gastric normal tissues (*n* = 13) and gastric tumor by TNM stages (stage I, *n* = 26; II, *n* = 110; III, *n* = 98; and IV, *n* = 14) from TCGA database using an Infinium Human Methylation 450 BeadChip. Statistical analysis of (**a**) and (**b**) were performed by *t*-test at *P* < 0.05 (*) or *P* < 0.001 (**) compared to normal gastric tissues. **c** Negative correlation between gene expression (RNA-seq) and promoter methylation (450K array) of each gene in gastric tumors (*n* = 230) from TCGA database. Statistical analysis was performed by Pearson’s correlation test

We further examined the expression of these six GI hormone receptors in 175 paired clinical tissues from the Chungnam National University Hospital (CNUH) cohort and found that these receptors were greatly downregulated in the primary GCs (paired *t*-test, *P* *=* 0.009 ~ 1.655 × 10^−9^) (Fig. 5c). We also investigated the clinical relevance of these six genes using a public database including 272 gastric tumors and 29 normal controls from TCGA cohort^23^. The RNA-seq data showed that the expression of the *PPYR1*, *PTGER2*, and *PTGER3* genes was significantly decreased during the TNM stage of gastric tumors compared to that in normal gastric tissues and that the expression of the remaining three genes tended to be decreased, but not statistically significant, in gastric tumors (Fig. 6a).

The Infinium Human Methylation 450 BeadChip data from TCGA also showed that the CpGs at the promoters of the five genes, except for the *PTGER2* gene, were heavily methylated during all TNM stages, especially demonstrating that the CpG methylation level was the highest during early stage and tended decrease along with GC progression (Fig. 6b). Moreover, the promoter methylation of five targets, except for *PPYR1*, was also significantly correlated to the expression of each gene (*R* = −0.291 ~ −0.448) (Fig. 6c).

### Molecular signatures of the six GI hormone receptors are highly informative regarding GC patient prognosis

**Fig. 7:**
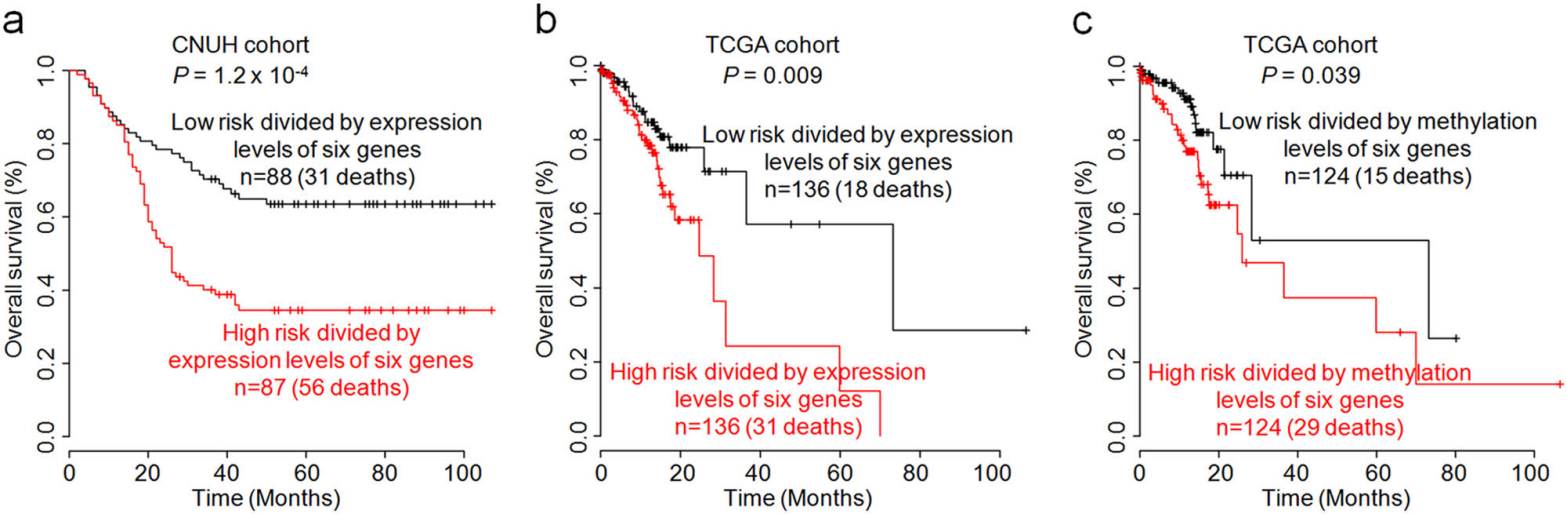
Survival analysis based on the expression data of the six GI hormone receptors in **(a)** CNUH and **(b)** TCGA cohorts and the methylation levels in TCGA cohort (**c**). All statistical analyses were performed by the Kaplan–Meier method and log-rank test

To assess the prognostic value of the six GI hormone receptors, a survival analysis was performed in multiple patient cohorts. The expression data of the six GI hormone receptors by qRT-PCR were adopted to create a risk score classifier, which was subsequently used as a risk assessment tool for GC prognosis in the CNUH cohort. The risk score of each patient was estimated using the regression coefficient of each of the six GI hormone receptors. Using a median cut-off risk score, the patients were divided into two groups (i.e., high- or low-risk groups). The survival rates significantly differed between the two groups in a log-rank test (*P* *=* 1.2 × 10^−4^; Fig. 7a). To validate the risk scoring system, a similar approach was directly applied to the RNA-seq gene expression data from TCGA cohort to dichotomize the patients into high-risk and low-risk groups. The Kaplan–Meier analysis revealed significant differences in patient survival between the two subgroups (log-rank test, *P* = 0.009; Fig. 7b). Based on the same procedure applied to the gene expression data, an additional approach was also applied to the six CpG methylation data sets (Fig. 7c) from TCGA cohort. By comparing the survival of two risk subgroups, the high-risk patient subgroup showed a significantly poorer prognosis than the low-risk subgroup (*P* *=* 0.039; Fig. 7c), although the predictive power of identifying the high-risk patients was clearly lower than that of the expression signature of the six genes. Thus, we established a proof-of-concept survival prediction panel consisting of six GI hormone receptors for identifying GC patients who have a high risk of survival.

## Discussion

To assess the molecular signatures and etiological causes of human cancer, the isolation of pure targeted cells from sequential stages of cancer development, such as normal, premalignant or cancer cells, is an important step. Using LCM technology, we successfully obtained an average of 1.22 × 10^5^ pure targeted cells from three sequential stages of GM, IM, and GT from a patient and generated methylome data using two different technologies, i.e., MBD-seq and RRBS.

Here, we summarize the DMR distribution in the IM and GT cell genomes compared to that in GM cells based on MBD-seq data as follows. First, a positive correlation between methylation enrichment and chromosomal aneuploidy was detected in the GT cells, indicating that CNV effects on methylation enrichment should be excluded in methylome analyses. After dividing the genome into three major regions, including the promoter, intragenic and intergenic regions, we found that the hypermethylated DMRs were highly enriched in CGIs in all three regions. Third, we found DMRs in the CGI shore and shelf regions surrounding CGIs and promoter CGIs, suggesting that DMRs play a significant role in the regulation of gene expression as previously described^24^. Fourth, we also found that repeat elements, such as LINEs, SINEs, LTRs, and satellite DNA, are predominant targets of hypomethylated DMRs in intergenic regions in the GT cell genome. This finding suggests that epigenetic mechanisms repress the activities of transposable elements, such as LINEs, and can be disrupted by demethylation in cancer cells, thus facilitating mutagenic retrotranspositions; this finding has also been shown in a previous report highlighting their potential impact in tumorigenesis^25^. Finally, we found that a small fraction (17%) of DMPs detected in GT cells was observed in IM cells, which are precancerous cells in gastric carcinogenesis, suggesting that aberrant methylation may be an early and essential step during gastric carcinogenesis^26^.

We focused on DMPs to reveal the critical pathway associated with gastric carcinogenesis. Using a gene set linked to the 3,035 DMPs derived from both the MBD-seq and RRBS data, the KEGG pathway analysis demonstrated that 59 genes linked to hypermethylated DMPs were significantly enriched in a neuroactive ligand-receptor interaction pathway; these genes, all of which code GPCRs, were associated with physiological homeostasis in the stomach, including the regulation of gastric acid secretion, gastric injury healing, smooth muscle contraction, etc. Because there are 256 members in the neuroactive ligand-receptor interaction pathway (GSEA, http://software.broadinstitute.org/gsea/), our study demonstrates that 23% (59/256) of the total entry into the pathway may be affected by epigenetic alterations in IM and/or GT cells. To the best of our knowledge, this report is the first to identify a unique pathway associated with human disease based on a methylome analysis. Here, we assert that the use of pure targeted cells could enable the identification of a specific pathway associated with gastric carcinogenesis.

Finally, we selected six GPCRs or GI hormone receptors as promising targets for GC treatment because they are functionally associated with the regulation of gastrin or gastric acid secretion, which plays a role in cell differentiation and carcinogenesis in the GI tract^27^. Gastric acid is secreted from parietal cells through the release of histamine from enterochromaffin-like (ECL) cells by gastrin produced from G cells in the gastric antrum^28,29^. In addition, gastrin has been shown to directly stimulate gastric acid release from parietal cells^30,31^. Subsequently, somatostatin (SST), which is secreted by D cells in the antrum in response to luminal acid, inhibits gastrin release in G cells by interacting with *SSTR2*, which encodes a receptor for SST. In the fundus, the release of SST by D cells in response to neurohumoral agents mediates the direct and indirect inhibition of gastric acid secretion in parietal cells by reducing ECL-cell histamine-release through SSTR2^27^. Thus, SSTR2 and SST, play an inhibitory role in gastrin-stimulated gastric acid secretion^32^. Furthermore, the loss of SST or SSTR2 inhibitory function in the stomach could lead to hypergastrinemia. Because the epigenetic silencing of *SST* has been described in GC^33,34^, notably, the epigenetic silencing of *SSTR2* might accelerate hypergastrinemia in the stomach.

The other GI hormone receptors, i.e., *PTGER2*, *PTGER3*^35^, *NPY1R*, and *PPYR1*^36^, also play inhibitory roles in gastric acid secretion, although the precise mechanisms are not fully understood. *PTGER2* and *PTGER3* encode receptors for prostaglandin E2 (PGE2) that inhibit gastric acid secretion in both parietal and ECL cells in the stomach^35^. It has been shown that the methylation of *PTGER2* is associated with neuroblastoma progression^37^ and prognosis in non-small cell lung cancer^38^. *PTGER3* methylation was detected in colorectal cancer (CRC)^39^ and gastric noninvasive neoplasia^40^. *NPY1R* and *PPYR1* encode a transmembrane protein that mediates the function of neuropeptide Y, which is a neurotransmitter, and peptide YY, which is a GI hormone, and both play inhibitory roles in gastric acid secretion^41^, tumor growth, and inflammation^42^. Epigenetic alterations have never been described in *NPY1R* and *PPYR1* in any type of human cancer thus far.

*PTGDR* encodes a receptor for prostaglandin D2 (PGD2) that plays protective roles against inflammatory changes in *H. pylori*-induced gastritis^43^. A recent report has shown that the *PTGDR* promoter is significantly methylated and associated with adenoma-carcinoma formation leading to CRC^44^. In this study, we found that *PTGDR* was methylated in the IM-GT sequences, suggesting that *PTGDR* methylation may be useful as an early detection biomarker in the development of GC and CRC.

While TCGA project provided a comprehensive catalog of driver genes for GC^23^, the sequence of genomic or epigenomic events that characterize the progression of precancerous lesions to advanced gastric cancer (AGC) remains to be unraveled. In this study, a comprehensive profiling of epigenomic changes that occur longitudinally in precancerous lesions or EGC as they progress towards AGC was provided and could be used to identify novel targets for GC interception that can be used to both develop early detection biomarkers and enable personalized therapeutic approaches. This approach may be a part of the “Pre-Cancer Genome Atlas (PCGA)”, which is a concerted initiative that has been recently proposed to characterize the molecular alterations in premalignant lesions and the corresponding changes in the microenvironment associated with progression to invasive carcinoma^45^.

Taken together, we detected epigenomic changes in gene promoter regions in intestinal-type EGC, and a small fraction (17%) was observed in IM, i.e., a precancerous lesion of the EGC. Overall, we showed that six GI hormone receptor genes may be targets for GC interception because their silencing by epigenetic alteration could lead to the dysregulation of gastrin or gastric acid secretion and thus progression to AGC, although the mechanism has not been fully elucidated. In addition, the expression panel of the six GI hormone receptors showed value as a prognostic factor. Finally, we suggest novel targets for GC interception that can be used to develop early detection, treatment biomarkers or survival predictors that can be used to identify GC patients who have a high risk of survival. Further investigation is needed to determine whether these targets are promising targets for GC interception using methylation inhibitors^46^ or targeted demethylation by the CRISPR/dCas9 system^47^.

## Electronic supplementary material

Supplementary material

Supplementary Table S5

Supplementary Table S6

Supplementary Table S8

Supplementary Table S9

Supplementary Table S11

## Data Availability

The data generated as a part of this study are available at the Gene Expression Omnibus (GEO) under accession numbers GSE55153 (for CNV), GSE55157 (for MBD-seq), and GSE55159 (for RRBS).
