# Supplementary material for: Whole genome MBD-seq and RRBS analyses reveal that hypermethylation of gastrointestinal hormone receptors is associated with gastric carcinogenesis
Source: Exp Mol Med. 2018 Dec 3;50(12):156. doi: 10.1038/s12276-018-0179-x (PMC6277407; doi:10.1038/s12276-018-0179-x)
Supplement: Supplementary file 1 — Supplementary material [file 12276_2018_179_MOESM1_ESM.docx]

Supplementary online material:

**Whole genome MBD-seq and RRBS analyses reveal that hypermethylation of gastrointestinal hormone receptors is associated with gastric carcinogenesis**

Hee-Jin Kim1*, Tae-Wook Kang1*, Keeok Haam1, Mirang Kim2,3, Seon-Kyu Kim1, Seon-Young Kim1,3, Sang-Il Lee4, Kyu-Sang Song5, Hyun-Yong Jeong6, Yong Sung Kim1,3^†^

1Genome Editing Research Center, 2Personalized Genomic Medicine Research Center, Korea Research Institute of Bioscience and Biotechnology (KRIBB), 125 Gwahak-ro, Yuseong-gu, Daejeon 34141, Republic of Korea, 3Department of Bioscience, Korea University of Science and Technology (UST), 217 Gajeong-ro, Yuseong-gu, Daejeon 34113, Republic of Korea, Department of 4General Surgery, ^5^Pathology, 6Internal Medicine, College of Medicine, Chungnam National University, 266 Moonwha-ro, Joong-gu, Daejeon 350157, Republic of Korea

*These authors contributed equally to this work.

^†^Correspondence and requests for reprints to: Yong Sung Kim, Genome Editing Research Center, KRIBB, 125 Gwahak-ro, Yuseong-gu, Daejeon 34141, Republic of Korea. Phone: +82-42-879-8110; Fax +82-879-8119; E-mail: [yongsung@kribb.re.kr](mailto:yongsung@kribb.re.kr)

**Short Running Head**: Hypermethylation of GI hormone receptor in GC

**Supplementary Materials and Methods**

**Multiple displacement amplification**

MDA was performed using the REPLI-g Midi Kit (Qiagen) for CNV analysis. Briefly, 10 ng of LCM-DNA was mixed with a master mix containing the reaction buffer, hexamer primers, and DNA polymerase in a 50 μl final volume reaction. Then, the mixed DNA was incubated at 30°C for 6 hours, followed by incubation at 65°C for 3 minutes to inactivate the enzyme. The yield of amplified DNA was estimated using the PicoGreen dye assay and real-time quantitative PCR.

**CNV analysis**

CNV analysis was performed in duplicate using 500ng of the MDA-DNA from the GM, IM and GT cells on the Affymetrix Genome-Wide Human SNP Array 6.0. After washing and staining, the arrays were scanned for data analysis using the Affymetrix GeneChip Command Console (AGCC) software. Genotypes were called by the Affymetrix Genotyping Console 3.0.1 (GTC) based on the Birdseed v2 algorithm. We analyzed the CNVs and LOH using the Chromosome Copy Number Analysis Tool (CNAT). Copy number changes and p-values were calculated based on the SNP hybridization signal intensity data from the experimental sample relative to the intensity distributions derived from a reference set containing over 100 ethnically diverse individuals. CNVs in the GT cells were estimated by comparing the normalized intensity for the SNP to the expected intensity for two chromosomes in the GM. LOH was searched by comparing the relative allele signals for each SNP between the GT and GM cells. As a heterozygous SNP in the GM was detected as a homozygous SNP in the GT, the site was considered as a LOH.

**Pyrosequencing analysis**

To estimate the content of the tumor cells in GT cells isolated by LCM, genotyping was performed in triplicate at 5 LOH loci, including rs2375874, rs1030465, rs10097518, rs2251110, and rs16952260, in the LCM-MDA and bulk DNA from the same patient used for the pyrosequencing analysis. Standard pyrosequencing was performed using the PSQ HS 96A system (Biotage AB) and the Pyro Gold Reagent Kit (Biotage AB) following the manufacturer’s instructions. Finally, SNP genotyping was performed automatically using the PyroMark analysis software (Biotage). PCR and sequencing primers for pyrosequencing were designed with PSQ Assay Design v1.0.6 (Biotage, Kungsgatan, Sweden; Supplementary Table S12).

**Supplementary Results**

**Correlation between methylation enrichment and chromosomal aneuploidy**

Because the GT cells greatly harbored CNVs in many chromosomes (Supplementary Fig. S3c), we examined the effects of CNVs on the enrichment of DNA methylation. The scatter plot patterns for the GM versus IM were very similar in all the chromosomes, and all the slopes were approximately 1 (Supplementary Fig. S3d). In contrast, the slopes in the plot for GT versus IM (Supplementary Fig. S3e) or GT versus GM (Supplementary Fig. S3f) were one-sided and slanted towards the *y*-axis. This finding indicates that DNA methylation was greatly enriched across the GT cell chromosomes compared to the IM and GM cells. Unusual methylation enrichment patterns were detected in the aneuploidy chromosomes of the GT cells, such as mono-, tri-, or tetra-ploidy, showing a direct correlation between methylation enrichment and chromosomal aneuploidy. These data are in agreement with previous findings reporting that DNA methylation data and genome coverage is highly dependent on the enrichment method employed [Robinson et al., 2010]. To account for the effects on methylation enrichment by aneuploidy, we normalized the read counts from the GT cell MBD-seq data taking into account the CNV effect. For example, because the long arm of chromosome 8 was tetraploid in the GT cells, the original GT MES was adjusted by dividing by two (Supplementary Fig. S5).

Robinson MD, Stirzaker C, Statham AL, Coolen MW, Song JZ, Nair SS *et al.* Evaluation of affinity-based genome-wide DNA methylation data: effects of CpG density, amplification bias, and copy number variation. *Genome Res* 2010; **20**(12)**:** 1719-29.

**Supplementary Tables**

| **Table S1**. Estimation of cell numbers and DNAs isolated by LCM procedure | | | |
| --- | --- | --- | --- |
| Tissue | Source | Cell number | DNA (ng) |
|  |  |  |  |
| Frozen tissues | GM | 123,640 | 860 |
|  | IM | 131,860 | 975 |
|  | GT | 111,550 | 1,155 |

| **Table S2**. Copy number variations detected in GT genome. | | | | | |
| --- | --- | --- | --- | --- | --- |
| Chromosome | Start | End | Size  (Mb) | Cytoband | CNV  (No. copy) |
| 2 | 7,578,209 | 218,782,798 | 211.2 | 2p25.1-q35 | Gain (3N) |
| 3 | 101,653,243 | 198,571,964 | 96.9 | 3q12.2-29 | Gain (3N) |
| 4 | 58,809 | 189,890,618 | 189.8 | 4p16.3-q35.2 | Loss (1N) |
| 5 | 95,189,205 | 132,400,117 | 37.2 | 5q15-31.1 | Loss (1N) |
| 6 | 5,733,364 | 58,812,024 | 53.1 | 6p25.1-11.1 | Gain (3N) |
| 7 | 64,619,667 | 76,276,456 | 11.7 | 7q11.21-23 | Gain (3N) |
| 8 | 292,394 | 41,600,253 | 41.3 | 8p23.3-11.21 | Loss (1N) |
| 8 | 42,036,597 | 146,268,947 | 104.2 | 8p11.21-q24.3 | Gain (4N) |
| 13 | 18,296,541 | 114,126,487 | 95.8 | 13q11-34 | Gain (4N) |
| 15 | 20,433,681 | 99,707,078 | 79.3 | 15q11.2-26.3 | Loss (1N) |
| 16 | 6,637,563 | 53,963,185 | 47.3 | 16p13.3-q12.2 | Gain (4N) |
| 16 | 68,640,862 | 74,200,352 | 5.6 | 16q22.1-23.1 | Loss (1N) |
| 18 | 184,118 | 75,742,145 | 75.6 | 18p11.32-q23 | Loss (1N) |
| 20 | 9,294 | 59,884,538 | 59.9 | 20p13-q13.33 | Gain (4N) |

| **Table S3**. Summary for MBD-seq and RRBS output. | | | | | | |
| --- | --- | --- | --- | --- | --- | --- |
| Tissue | Method^a^ | Samples | No. lanes | No. total reads^b^ (x10^6^) | No. aligned reads (x10^6^) | Alignment rate (%) |
| LCM tissues | MBD-seq | GM_LCM | 1 | 24.3 | 17.1 | 70.4 |
|  |  | IM_LCM | 1 | 28.3 | 18.6 | 64.7 |
|  |  | GT_LCM | 1 | 24.6 | 17.2 | 69.9 |
|  | RRBS | GM_LCM | 2^c^ | 13.0 | 8.1 | 62.4 |
|  |  | IM_LCM | 1 | 18.9 | 7.2 | 37.9 |
|  |  | GT_LCM | 1 | 13.4 | 5.4 | 40.0 |
| ^a^ All sequencing was performed in 2010 using the Illumina Genome Analyzer II (76-bp, single reads). | | | | | | |
| ^b^ Total read numbers per lanes averaging ~25 million for MBD-seq and close to ~15 million for RRBS.  ^c^ RRBS for GM sample was carried out twice due to a small amount. Because correlation of two experiments for GM was shown to be almost identical pattern (*Pearson*'s correlation, *r* = 0.98, *p*-value < 2.2e-16), the reads from two experiments were summed for further analysis. | | | | | | |

| **Table S4**. Summary for DMPs identified from MBD-seq data. | | | | | | |
| --- | --- | --- | --- | --- | --- | --- |
| Group | Hypermethylated | |  | Hypomethylated | |  |
|  | DMRs | DMPs |  | DMRs | DMPs |  |
| Early-onset | 3,503 | 193 |  | 7,592 | 151 |  |
| GT-specific | 36,528 | 1,749 |  | 41,046 | 668 |  |
| Total | 40,031 | 1,942 |  | 48,638 | 819 |  |

**Table S5.** Number of genes associated with hypermethylated DMPs from MBD-seq data. (MS Excel file)

**Table S6.** Number of genes associated with hypomethylated DMPs from MBD-seq data. (MS Excel file)

| **Table S7**. Summary for DMPs identified from RRBS data. | | | | | | |
| --- | --- | --- | --- | --- | --- | --- |
| Group | Hypermethylated | |  | Hypomethylated | |  |
|  | DMRs | DMPs |  | DMRs | DMPs |  |
| Early-onset | 20,209 | 144 |  | 22,534 | 83 |  |
| GT-specific | 26,727 | 359 |  | 26,257 | 91 |  |
| Total | 46,936 | 503 |  | 48,791 | 174 |  |

**Table S8.** Number of genes associated with hypermethylated DMPs from RRBS data. (MS Excel file)

**Table S9.** Number of genes associated with hypomethylated DMPs from RRBS data. (MS Excel file)

| **Table 10.** The number of DMPs identified from MBD-seq and RRBS data set. | | | | | | | | | | | |
| --- | --- | --- | --- | --- | --- | --- | --- | --- | --- | --- | --- |
| Methods | Hypermethylated DMPs | | | | |  | Hypomethylated DMPs | | | | |
|  | Early-  onset | % | GT-specific | % | Total |  | Early-  onset | % | GT-specific | % | Total |
| MBD-seq | 193 | 10 | 1749 | 90 | 1942 |  | 151 | 18 | 668 | 82 | 819 |
| RRBS | 144 | 29 | 359 | 71 | 503 |  | 83 | 48 | 91 | 52 | 174 |
| Combined | 289 | 14 | 1792 | 86 | 2081 |  | 225 | 24 | 729 | 76 | 954 |

**Table S11.** Putative identification of genes linked to DMPs from the combined MBD-seq and RRBS data. (MS Excel file)

**Table S12**. Primer sequences used in this study

| Target | Primer sequences | Tm (℃) | Cycles | Product (bp) |
| --- | --- | --- | --- | --- |
| a) Primer sequences used for genotyping of pyrosequencing | | | | |
| rs2375874 | F: 5'-GCATTCAGGTTTCTTTGATCTTAC-3' | 60 | 35 | 89 |
|  | R: 5'-Biotin-ATGGCCTCTACTTAAAGCTATTCA-3' |  |  |  |
|  | S: 5'-GGTTTCTTTGATCTTACACC-3' |  |  |  |
| rs1030465 | F: 5'-AAGGGCATGTATCTTAGTGACCA-3' | 55 | 35 | 212 |
|  | R: 5'-Biotin-TCCACTTTTCGAGAATGTTCTG-3' |  |  |  |
|  | S: 5'-CAAAATGGACACTGCTT-3' |  |  |  |
| rs10097518 | F: 5'-CTATTTCTGGGCATGCTTC-3' | 60 | 35 | 120 |
|  | R: 5'-Biotin-AGAAGGCAGAAATGGTTGT-3' |  |  |  |
|  | S: 5'-GCTTCATGCATTCACAC-3' |  |  |  |
| rs2251110 | F: 5'-Biotin-TGGTATGGCTTCCAAGTTTTA-3' | 55 | 35 | 162 |
|  | R: 5'-CTGCTTTTAATTTGGTGTCAGTAG-3' |  |  |  |
|  | S: 5'-TTAATTTGGTGTCAGTAGG-3' |  |  |  |
| rs16952260 | F: 5'-ATGGATTTCTTTTCATTTCAGATCTA-3' | 55 | 35 | 96 |
|  | R: 5'-Biotin-TAAGGCTGTTTCTATTTGTAAATAAGTT-3' |  |  |  |
|  | S: 5'-TTCATTTCAGATCTATTGTT-3' |  |  |  |
| b) Primer sequences used for RT-PCR and qRT-PCR | | | | |
| *ADCYAP1R1* | F: 5'-CAGCAAAAGGGAAAGACTCG-3' | 62 | 35 | 151 |
|  | R: 5'-CACAGCGAAGTAACGGTTCA-3' |  |  |  |
| *APLNR* | F: 5'-CACCATCATGCTGACCTGTT-3' | 62 | 35 | 245 |
|  | R: 5'-AGGTGCAGTAGGGGAAGATG-3' |  |  |  |
| *CALCR* | F: 5'-CTGGGAATCCAGTTTGTCGT-3' | 62 | 33 | 194 |
|  | R: 5'-CGCTGGTTCCACTGAATTTT-3' |  |  |  |
| *DRD4* | F: 5'-CCTTCCTGCTGTGCTGGAC-3' | 68 | 35 | 160 |
|  | R: 5'-AACTCGGCGTTGAAGACAGT-3' |  |  |  |
| *GALR1* | F: 5'-TTCGCAAAGATTCACACCTG-3' | 64 | 35 | 179 |
|  | R: 5'-CAAGTTGCAGCATCGCTTAC-3' |  |  |  |
| *GHSR* | F: 5'-GCTCCTTGGAGATTGCTCAG-3' | 64 | 35 | 150 |
|  | R: 5'-AGGGTTCGAATCCCAGAAGT-3' |  |  |  |
| *MTNR1B* | F: 5'-TGGTCATCCACTTCCTCCTC-3' | 68 | 35 | 190 |
|  | R: 5'-TTAAGTGGAGCCCAGCAGAT-3' |  |  |  |
| *NPY1R* | F: 5'-TGCTACCTGCAACCACAATC-3' | 60 | 35 | 202 |
|  | R: 5'-GGAAACATCTGTGTGCATCG-3' |  |  |  |
| *NPY5R* | F: 5'-CTGGCAGCCATAAATGGAGT-3' | 62 | 35 | 187 |
|  | R: 5'-GGGACCCCTGGTATGAACTT-3' |  |  |  |
| *NTSR1* | F: 5'-ACCATCAACCCCATCCTGTA-3' | 68 | 35 | 190 |
|  | R: 5'-CAGCCTAGTACAGCGTCTCG-3' |  |  |  |
| *PPYR1* | F: 5'-GGCGGATAAGGTGGTCTGTA-3' | 62 | 33 | 209 |
|  | R: 5'-CATTGACCTGCTTCATGTGC-3' |  |  |  |
| *PTGDR* | F: 5'-CGAGCCTTGCGATTTCTATC-3' | 62 | 35 | 150 |
|  | R: 5'-TTCCATGTTAGTGGAATTGCTG-3' |  |  |  |
| *PTGER2* | F: 5'-GCCAGTAAACAGGCTGACCT-3' | 68 | 35 | 197 |
|  | R: 5'-CATGAAGTGCCTTGTCAGCA-3' |  |  |  |
| *PTGER3* | F: 5'-AGCTTATGGGGATCATGTGC-3' | 60 | 33 | 198 |
|  | R: 5'-ACAGCAGGTAAACCCAAGGA-3' |  |  |  |
| *SSTR2* | F: 5'-CCCCTCACCATCATCTGTCT-3' | 62 | 33 | 247 |
|  | R: 5'-AGGTGAGGACCACCACAAAG-3' |  |  |  |
| *SSTR4* | F: 5'-TCTTTGTGCTCTGCTGGATG-3' | 64 | 35 | 180 |
|  | R: 5'-AGAGAACCCGCTGGAAGAAT-3' |  |  |  |
| *VIPR2* | F: 5'-ACGGAGACCTCGGTCATCTA-3' | 66 | 35 | 163 |
|  | R: 5'-GACAACCAGCTTGACGGAGT-3' |  |  |  |
| *B-actin* | F: 5'-CAAGAGATGGCCACGGCTGCT-3' | 68 | 25 | 283 |
|  | R: 5'-TCCTTCTGCATCCTGTCGGCA-3' |  |  |  |
| c) Primer sequences used for bisulfite sequencing | | | | |
| *NPY1R* | F: 5'-ATTTTTTTAGTTTATTTGGGAATTAATG-3' | 56 | 35 | 547 |
|  | R: 5'-ATAAAAATCCAAATTTTCCTTTACC-3' |  |  |  |
| *PPYR1* | F: 5'-TTTGTTTGTATTTAGGGTTGAGGT-3' | 62 | 35 | 494 |
|  | R: 5'-TACACCCCTTACCTCCTTAACTTAC-3' |  |  |  |
| *PTGDR* | F: 5'-TTTGGTGTTTTATTTAGATGGTTTA-3' | 62 | 35 | 346 |
|  | R: 5'-ACAATCTCAATACCCTACTACCTC-3' |  |  |  |
| *PTGER2* | F: 5'-GTTGTTGGATTATGGGTAGTA-3' | 56 | 35 | 436 |
|  | R: 5'-TACAAATTACAAAAAATTTATAAAAC-3' |  |  |  |
| *PTGER3* | F: 5'-TTAGGTTGTAGGAAAAGGTGATTGT-3' | 60 | 35 | 500 |
|  | R: 5'-CAAAAAATCCTTCCTACTATACATC-3' |  |  |  |
| *SSTR2* | F: 5'-GGGTTGGTTGGGTTAGTTTTAG-3' | 60 | 35 | 393 |
|  | R: 5'-CAAATACACACAAATACCCAAATAC-3' |  |  |  |

**Supplementary Figures**

**
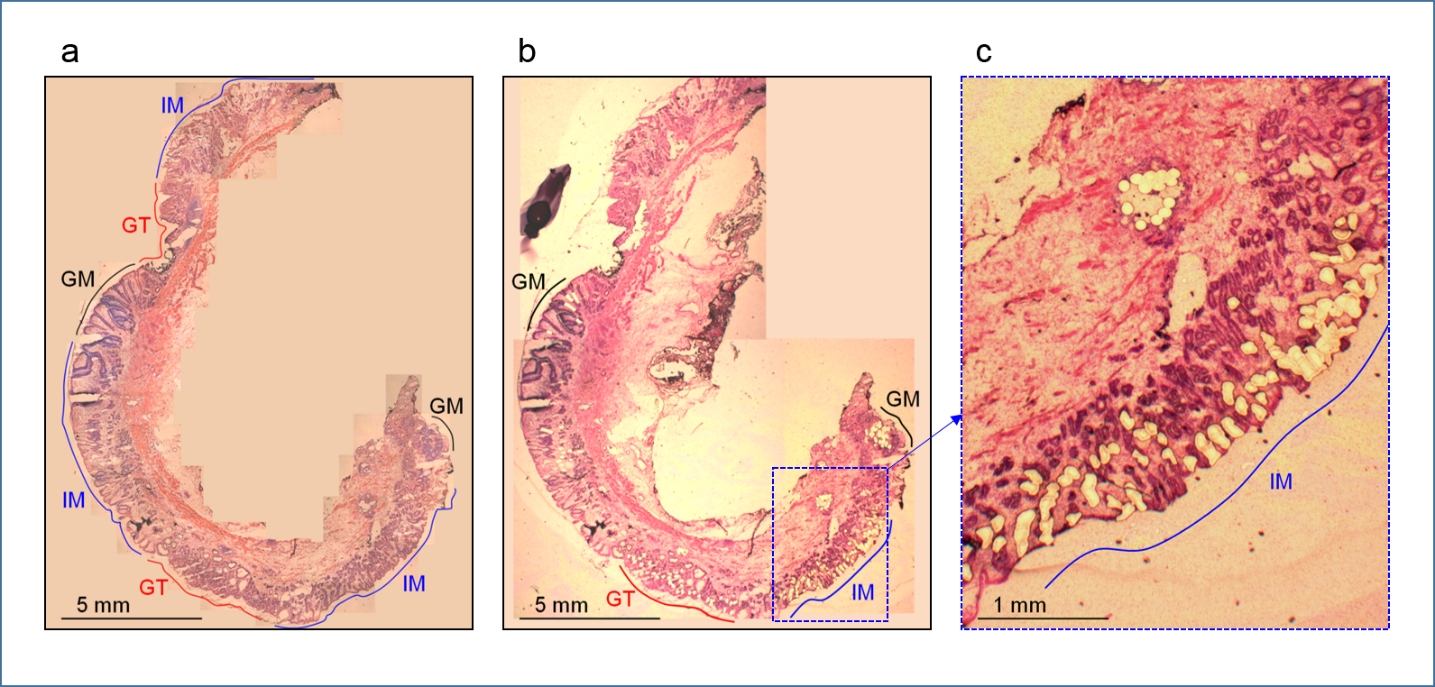
**

**Figure S1.** Isolation of GM, IM, and GT cells with high homogeneity from frozen ESD tissue from a patient with intestinal-type EGC via a LCM procedure. (a) and (b), photos before and after LCM. (c), an enlarged figure of blue-dotted tetragon after LCM.

**
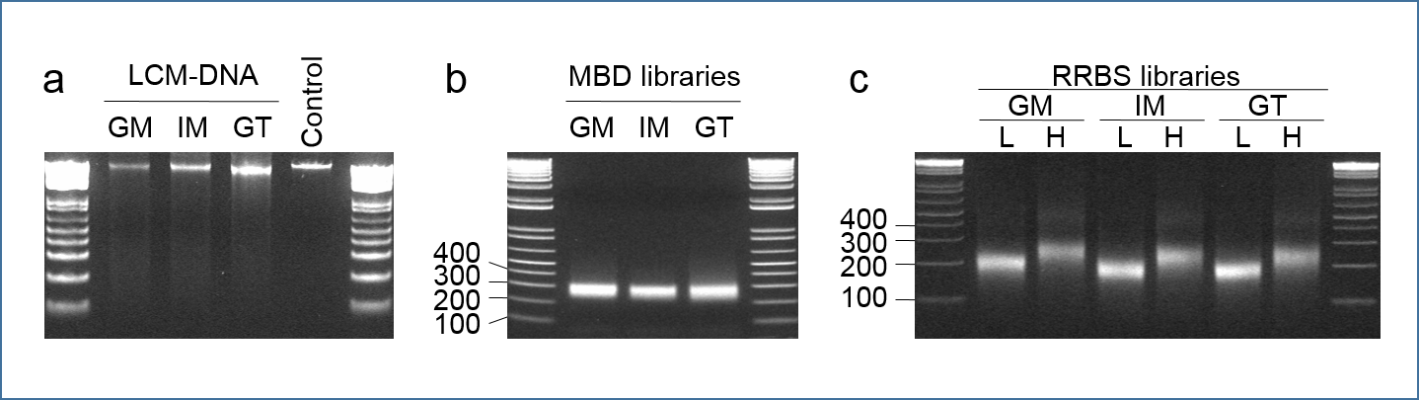
**

**Figure S2.** Agarose gel electrophoresis of the LCM-DNAs and sequencing libraries. (a) DNAs purified from GM, IM, and GT cells using LCM. (b) DNAs from the MBD-seq libraries. (c) DNAs from the RRBS libraries.

**
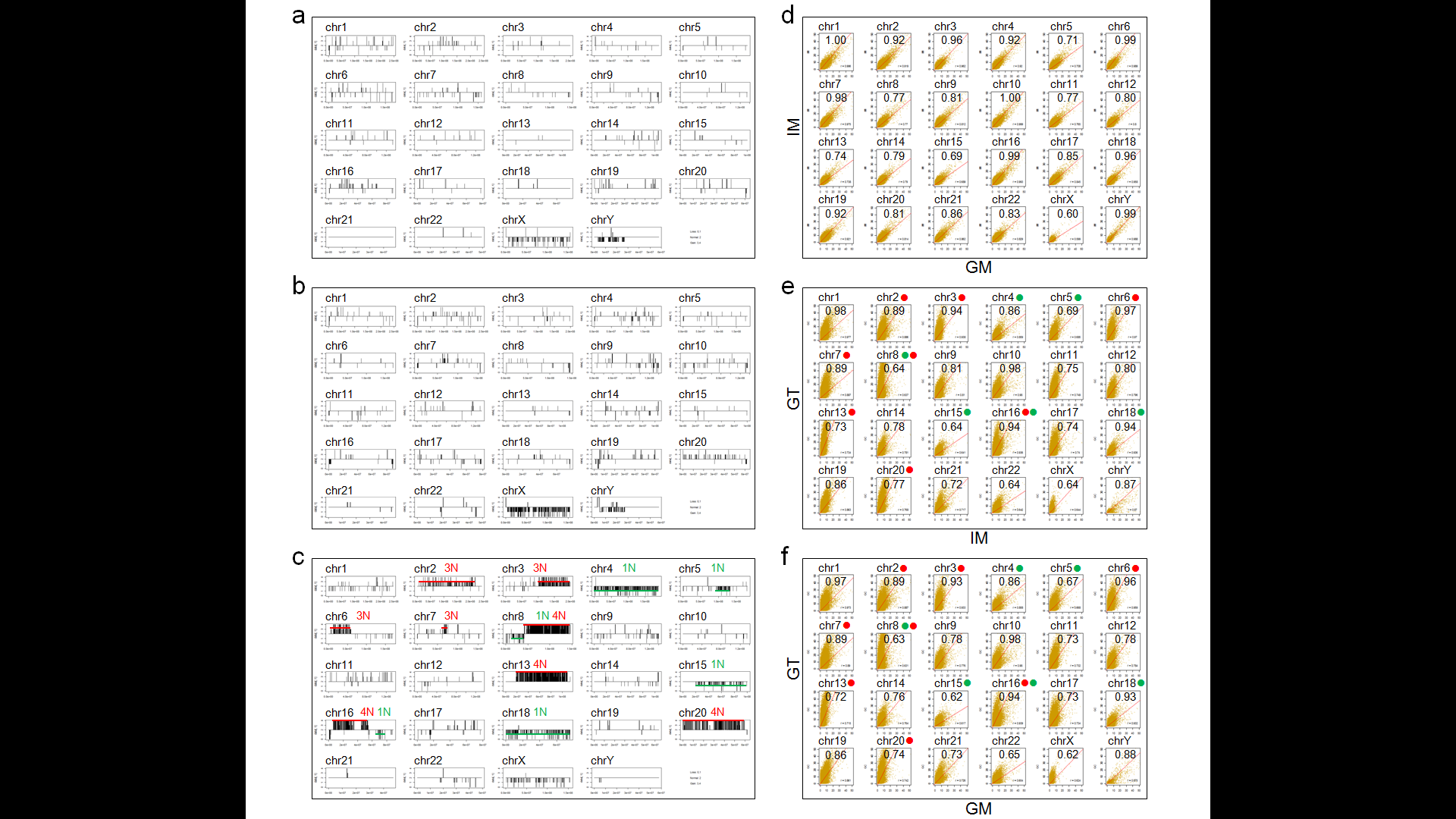
**

**Figure S3.** CNVs and MBD-seq analysis for GM, IM, and GT cells. (a, b, and c). Each SNP data was compared to the reference human data and illustrated using the CNAT. Lines with red and green colors indicate regions that chromosomal gain or loss were occurred in each chromosome. Scatter plot analysis of (d) GM versus IM, (e) IM versus GT, and (f) GM versus GT in each chromosome based on the MBD-seq data. All ratio from (d), (e), and (f) indicate Pearson's correlation coefficient. Red or green dots show chromosomal gain or loss in each chromosome corresponding to (c).

**
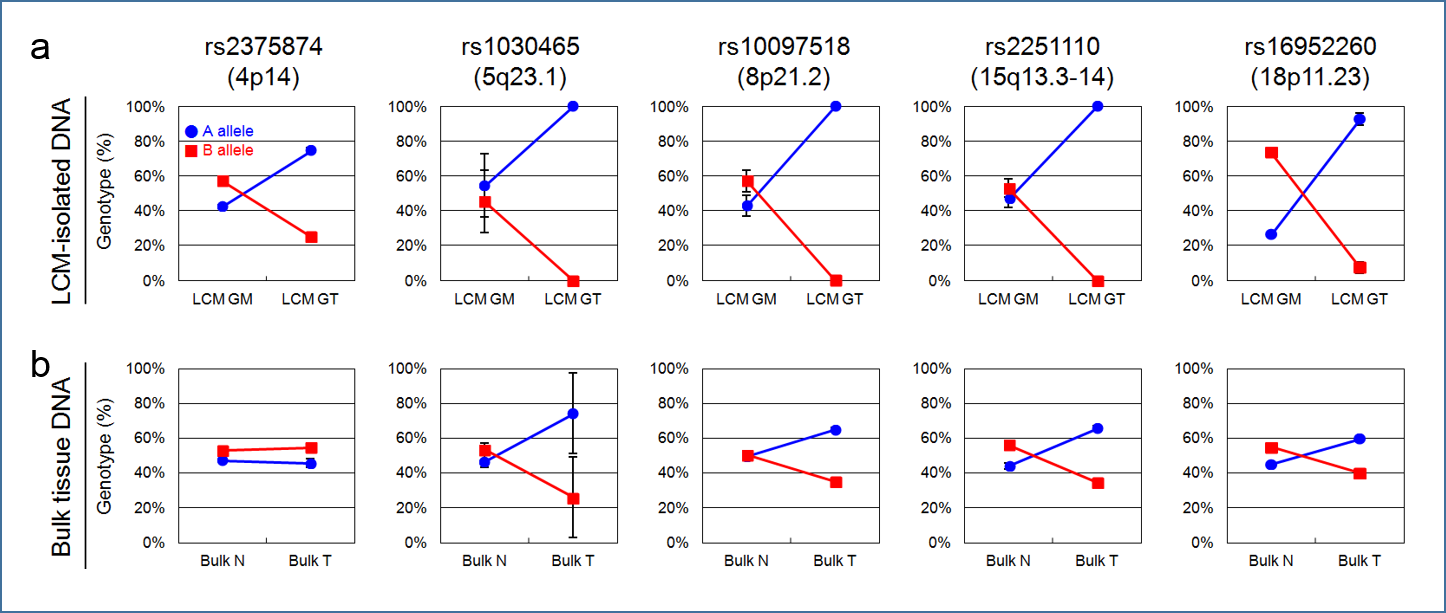
**

**Figure S4.** Cell homogeneity test for LCM-isolated cells. The genotypes of SNP markers at five LOH loci in GT, including rs2375874, rs1030465, rs10097518, rs2251110, and rs16952260, were compared between (a) LCM-isolated DNA and (b) bulk tissue DNA. The red ‘B’ allele indicates a minor allele when was lost in GT cell, while the blue ‘A’ allele means the remaining allele as a major allele.

**
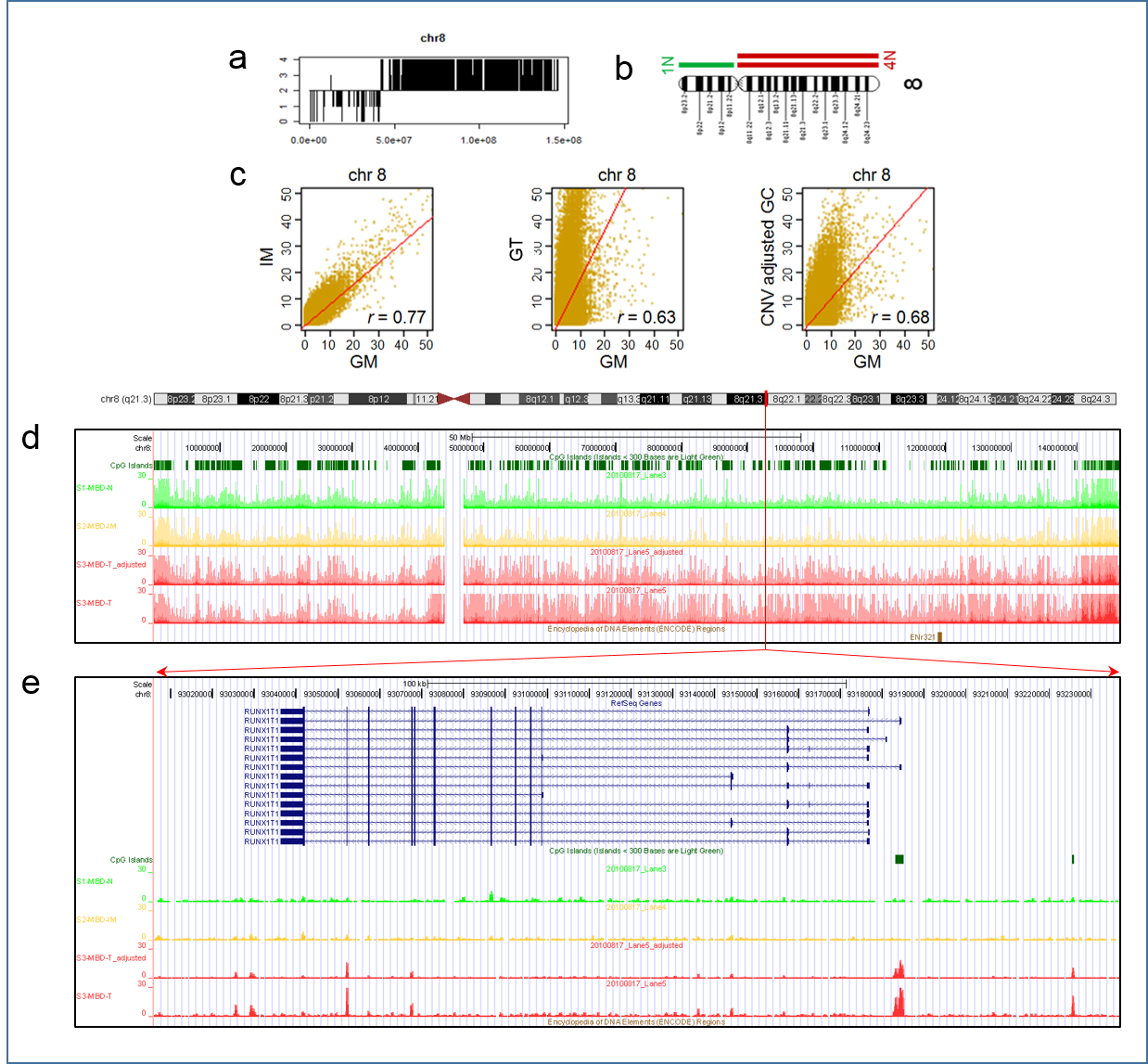
**

**Figure S5.** An example of normalization using methylation enrichment based on CNV analysis in the GT cell genome. (a) CNV output for GT cell chromosome 8. (b) Aneuploidy of chromosome 8 in GT cells. (c) Scatter plot analysis of GM versus IM (left), GM versus GT (middle), and GM versus GT adjusted based on CNVs (right). (d) Diagram of methylation enrichment on chromosome 8 of GM (1st lane), IM (2nd lane), and GT, which are adjusted based on CNV data (3rd lane) and GT (4th lane). (e) An enlarged diagram of normalized enrichment for a specific genomic region in chromosome 8.
